# Characterization of a minimal pKW2124 replicon from *Weissella cibaria* KLC140 and its application for the construction of the *Weissella* expression vector pKUCm1

**DOI:** 10.3389/fmicb.2015.00035

**Published:** 2015-02-03

**Authors:** Hye-Jin Ku, Myeong Soo Park, Ju-Hoon Lee

**Affiliations:** ^1^Department of Food Science and Biotechnology, Institute of Life Sciences and Resources, Kyung Hee UniversityYongin, South Korea; ^2^Department of Hotel Culinary Arts, Yeonsung UniversityAnyang, South Korea

**Keywords:** *Weissella*, minimal replicon, expression vector, β-galactosidase, theta replication

## Abstract

A 2.1-kb plasmid was previously isolated from *Weissella cibaria* KLC140 in kimchi and cloned into pUC19 along with the *slpA* and *gfp* genes, resulting in an 8.6-kb pKWCSLGFP construct for use as a novel surface display vector. To reduce the size of the vector, the minimal replicon of pKW2124 was determined. The pKW2124 plasmid contains a putative origin of replication *(ori*), a potential ribosomal binding site (RBS), and the *repA* gene encoding a plasmid replication protein. To conduct the minimal replicon experiment, four different PCR products (MR1, *ori*+RBS+*repA*; MR2, RBS+*repA*; MR2’, *repA*; MR3, fragment of *repA*) were obtained and cloned into pUC19 (pKUCm1, pKUCm2, pKUCm2’, and pKUCm3, respectively) containing the chloramphenicol acetyltransferase (CAT) gene. These constructed vectors were electroporated into *W. confusa* ATCC 10881 with different transformation efficiencies of 1.5 × 10^5^ CFU/μg, 1.3 × 10^1^ CFU/μg, and no transformation, respectively, suggesting that the putative *ori*, RBS, and *repA* gene are essential for optimum plasmid replication. Subsequent segregational plasmid stability testing of pKUCm1 and pKUCm2 showed that the vector pKUCm1 is highly stable up to 100 generations but pKUCm2 was completely lost after 60 generations, suggesting that the putative *ori* may be important for plasmid stability in the host strain. In addition, a host range test of pKUCm1 revealed that it has a broad host range spectrum including *Weissella, Lactococcus, Leuconostoc,* and even *Lactobacillus*. To verify the application of pKUCm1, the β-galactosidase gene and its promoter region from *W. cibaria* KSD1 were cloned in the vector, resulting in pKUGal. Expression of the β-galactosidase gene was confirmed using blue-white screening after IPTG induction. The small and stable pKUGal vector will be useful for gene transfer, expression, and manipulation in the *Weissella* genome and in other lactic acid bacteria.

## INTRODUCTION

Members of the genus *Weissella* are Gram-positive, non-spore forming, heterofermentative, non-motile, and short-rod bacteria belonging to the family *Leuconostocaceae* (Collins et al., 1993; Lee et al., 2002). Since *Weissella* was first characterized by Collins et al. (1993), this novel species has been continuously isolated from various sources including fermented foods (Lee et al., 2002; Magnusson et al., 2002; Tohno et al., 2013). To date, 14 species of *Weissella* are listed in the Taxonomic Outline of Bacteria and Archaea, release 7.7 (TOBA 7.7; Garrity et al., 2007). They are recognized as lactic acid bacteria (LAB) and generally produce lactic acid and various exopolysaccharides (EPSs), which are likely involved in food flavor and texture (Galle et al., 2010). Interestingly, novel bacteriocins (weissellicin 110 from *W. cibaria*, and weissellicin Y and M from *W. hellenica*) were reported in these strains, suggesting the potential for new food preservatives (Srionnual et al., 2007; Masuda et al., 2012). Furthermore, the *W. confusa* strain PL9001 inhibited *Helicobacter pylori*, suggesting that *Weissella* has the potential as a probiotic for promoting stomach health (Nam et al., 2002).

To explore the host beneficial effects of *Weissella*, its molecular and genomic study is required. Since the draft genome sequence of *W. cibaria* KACC 11862 was first reported in 2011 (Kim et al., 2011), the complete genome sequence of *W. koreensis* KACC 15510 (Lee et al., 2011) and the draft genome sequences of *W. thailandensis* fsh4-2 (Benomar et al., 2011), *W. koreensis* KCTC 3621^T^ (Lee et al., 2012), and *W. confusa* LBAE C39-2 (Amari et al., 2012) were reported and deposited in the GenBank database. However, only a few *Weissella* plasmids were sequenced to develop molecular tools to study their genomes, including pKLCA (1.5-kb), pKLCB (3.4-kb), pKLCC (10.0-kb), and pKW2124 (2.1-kb) from *W. cibaria* KLC140 (Park et al., 2007; Kim et al., 2013). In an effort to develop the first *Weissella* cloning and expression vector system, the 2.1-kb theta-replicating plasmid pKW2124 was characterized (Kim et al., 2013). To construct a *Weissella* cloning/expression vector, the pTSLGFP vector containing a fused *slpA* gene encoding a surface layer protein with the *gfp* gene was cloned into pKUWCAT containing the entire pKW2124 plasmid and the chloramphenicol acetyltransferase (CAT) gene, resulting in a 8.6-kb pKWCSLGFP vector (Kim et al., 2013). However, minimization of the vector is needed, via determination of the minimal replicon of pKW2124, to increase transformation efficiency and vector stability.

In this study, the small plasmid from *W. cibaria* KLC140, pKW2124, was dissected and tested to determine the minimal replicon. Using the minimal pKW2124 replicon, the stable *Escherichia coli-Weissella* shuttle cloning vector pKUCm1 was constructed and its host spectrum was studied. This minimalized expression vector was used for the cloning and heterologous expression of the β-galactosidase gene (pKUGal), indicating that this improved expression vector system will be a useful molecular tool for gene cloning and expression in *Weissella.*

## MATERIALS AND METHODS

### BACTERIAL STRAINS, PLASMIDS, PCR PRIMERS, AND GROWTH CONDITIONS

The bacterial strains, plasmids, and primers used in this study are listed in **Table 1**. All *Weissella* species were incubated anaerobically at 37°C in de Man-Rogosa-Sharpe (MRS) medium (Difco, Detroit, MI, USA) and *E. coli* DH5α (Invitrogen, Carlsbad, CA, USA) was grown with shaking in Luria-Bertani (LB) medium (Difco) at 37°C. Ampicillin sulfate (Sigma, St. Louis, MO, USA) was added to the *E. coli* growth medium for selection at 50 μg/ml. *Weissella* and other electroporation host bacteria were selected using chloramphenicol (USB Corporation, Santa Clara, CA, USA) with the following appropriate concentrations, 6.0 μg/ml for *Weissella, Lactobacillus* and *Bifidobacterium,* 5.0 μg/ml for *Lactococcus* and *Leuconostoc*, and 3.0 μg/ml for *Streptococcus*. Agar medium was prepared with supplementation of 1.8% Bacto agar (Difco).

**Table 1:** Bacterial strains, plasmids, and primers used in this study.

	Relevant characteristic(s)^a^	Source or reference
**Bacterial strains**		
* Escherichia coli* DH5α	Cloning host (*fhuA2 lac(del)U169 phoA glnV44 Φ80*′ *lacZ(del)M15 gyrA96 recA1 relA1 endA1 thi-1 hsdR17*)	Invitrogen
*Weissella cibaria* KLC140	Host of pKW2124	Kim et al. (2013)
*W. cibaria* KSD1	Source of β-galactosidase gene	DCC
*W. confusa* ATCC 10881	Electroporation host for host range and β-galactosidase gene expression; No β-galactosidase activity	ATCC
*Leuconostoc citreum* 75X	Electroporation host for host range	DCC
*Lactococcus lactis* subsp. *cremoris* MG1363	Electroporation host for host range	Wegmann et al. (2007)
*Lactobacillus plantarum* 50W	Electroporation host for host range	DCC
*W. paramesenteroides* KCTC 3531	Electroporation host for host range	KCTC
*Streptococcus thermophilus* ATCC 19258	Electroporation host for host range	ATCC
*Bifidobacterium bifidum* ATCC 15521	Electroporation host for host range	ATCC
**Plasmids**		
pKW2124	2.1-kb wild-type plasmid from *W. cibaria* KLC140	Kim et al. (2013)
pUC19	2.7-kb; *E. coli* cloning vector; Amp^r^	Thermo scientific
pC194	2.9-kb; *Staphylococcus aureus* plasmid; Cm^r^	Horinouchi and Weisblum (1982)
pUCm	3.7-kb; Cm^r^ cassette from pC194 in *Nde*I-*Bam*HI site of pUC19	This study
pKUCm-series	Set of *E. coli*-*W. confusa* shuttle vectors based on pUCm	This study
pMR1	2.7-kb; self-ligated MR1 (*W. cibaria* pKW2124 plasmid minimal replicon); Cm^r^	This study (See **Figure 1**)
**Primers (5′-3′)**
SA-CmF-NdeI/forward	CATATGAGAAAAGGATTTTTCGCTA	This study
SA-CmR-BamHI/reverse	GGATCCGATTATAGGTATGTGGTTTT	This study
WC-F1-XbaI/forward	TCTAGATTCTTATCTTGATACTATT	This study
WC-F2-XbaI/forward	TCTAGATGGATAAAACGGGAGGAGCT	This study
WC-F2’-XbaI/forward	TCTAGA ATGTCTGAAGTATTTAAAGATT	This study
WC-F3-XbaI/forward	TCTAGAGCTAACTAAGGCTTTTGGTA	This study
WC-R1-XbaI/reverse	TCTAGAATAAACATAACGCCAAACA	This study
pMR1-F1-PstI/forward	CTGCAGCTGGCTTAACTATGCGGC	This study
pMR1-R1-PstI/reverse	CTGCAGGAAACAGCTATGACCATGATT	This study
WC-proGalF-BamHI/forward	GGATCCGTGACTGATTTCTACTTGG	This study
WC-proGalR-BamHI/reverse	GGATCCGTGACTGATTTCTACTTGG	This study

*^a^Amp^r^ and Cm^r^, resistance to ampicillin and chloramphenicol, respectively; Underlined sequence in primers indicate introduced restriction enzyme sites (*Nde*I, *Bam*HI, *Xba*I, and *Pst*I)*.

*^b^ATCC, American Type Culture Collection; KCTC, Korean Collection for Type Culture; DCC, Dairy Culture Collection, Kyung Hee University*.

### PLASMID PREPARATION AND MOLECULAR TECHNIQUES

Plasmid preparation from all *Weissella* species was followed as previously described by O’Sullivan and Klaenhammer (1993). For plasmid preparation, bacterial cells of 15 ml overnight culture was harvested and resuspended in 1 ml of TES buffer (30 mM Tris HCl, pH 8.0, 50 mM NaCl, 5 mM EDTA, pH 8.0, final concentration). After pelleting, the cells were resuspended in 200 μl of sucrose-lysozyme solution (30 mg/ml lysozyme in 25% sucrose, 50 mM Tris HCl, pH 8.0, and 1 mM EDTA, pH 8.0, final concentration) and were incubated for 1 h at 37°C. After treatment with alkaline sodium dodecyl sulfate (SDS) solution (3% SDS in 0.2 N NaOH, final concentration) for 7 min at room temperature, 300 μl of pre-chilled 3 M sodium acetate (pH 4.8) was added and gently mixed. After centrifugation, the supernatant was mixed with a 650 μl of isopropanol and centrifuged to pellet plasmid DNA. The DNA pellet was resuspended in 320 μl of molecular-grade water (MW) with 2 μl of RNase A (100 mg/ml) and incubated for 1 h at 37°C. After RNase A treatment, the plasmid DNA was extracted using saturated phenol–chloroform/isoamyl alcohol and the water layer was transferred into sterilized 1.5 ml tube. The plasmid DNA was precipitated with ice-cold ethanol and resuspended with 20 μl of MW. Plasmid DNA from *E. coli* was isolated using the Axyprep Plasmid Miniprep Kit (Axygen Biosciences, Union City, CA, USA) according to the manufacturer’s instructions. The general procedures for restriction enzyme digestion and recombinant DNA preparation were carried out according to standard procedures (Sambrook et al., 2001). All PCR reactions were performed with KOD -Plus- high fidelity DNA polymerase (TOYOBO, Osaka, Japan) according to the manufacturer’s protocols.

### PLASMID TRANSFER

*Escherichia coli* DH5α (Invitrogen) cells were used as the competent strain for standard heat-shock transformation (Sambrook et al., 2001). The electroporation procedure and conditions for *W. confusa* ATCC 10881 are as follows. An overnight MRS broth culture of *W. confusa* ATCC 10881 was sub-inoculated in MRS broth containing 0.25 M sucrose and grown to an optical density of 0.5 at 600 nm. The cells were harvested by centrifugation and washed twice by re-suspension of the pellet with the same volume of ice-cold electroporation buffer (0.5 M sucrose and 10% glycerol). Then, the pellet was re-suspended in ice-cold electroporation buffer containing lysozyme (600 U ml^-1^, final concentration) and incubated at 37°C for 20 min. Cells were washed twice with ice-cold electroporation buffer and re-suspended in the small volume of buffer (1% of total culture volume). For electroporation, 50 μl of competent cells and 0.1 μg plasmid DNA were electroporated in a 2-mm pre-chilled cuvette at 25 μF, 400 Ω, and 2 kV cm^-1^. Cells were immediately re-suspended in 1.0 ml recovery medium (MRS broth supplemented with 0.5 M sucrose) and incubated at 37°C for 2 h. Transformants were selected using MRS agar containing 6.0 μg/ml chloramphenicol for 2 days at 37°C.

### SEGREGATIONAL STABILITY OF THE MINIMAL REPLICON

The segregational stability of the pKUCm series of plasmids harbored in *W. confusa* ATCC 10881 without antibiotic selection was monitored as described previously (Lee and O’Sullivan, 2006). The presence of plasmids was finally verified by plasmid preparations and subsequent restriction enzyme digestion.

### DETERMINATION OF pKUCm1 HOST RANGE

The electroporation host strains listed in **Table 1** were used for transformation with pKUCm1 and the colonies were selected against appropriate chloramphenicol concentrations. The selected colonies were used for plasmid re-isolation and restriction enzyme digestion for confirmation of the pKUCm1 host range.

### β-GALACTOSIDASE GENE CLONING, EXPRESSION, AND ASSAYS FOR ACTIVITY

The promoter region of the β-galactosidase gene was predicted using the BPROM program (Li, 2011). The β-galactosidase gene and its promoter region (ProGal) in *W. cibaria* KSD1 were PCR amplified with primers WC-proGalF-BamHI and WC-proGalR-BamHI (**Table 1**) using genomic DNA from *W. cibaria* KSD1 as a template. The PCR conditions were as follows: 94°C for 2 min followed by 30 cycles of 15 s at 94°C, 30 s at 52°C, 3 min at 68°C, and a final extension for 5 min at 68°C. The PCR product was purified, digested with *Bam*HI, and cloned into the *Bam*HI site of pKUCm1, resulting in pKUGal. The pKUGal plasmid was electroporated into *W. confusa* ATCC 10881 and β-galactosidase activity was confirmed by blue-white screening on MRS agar plates supplemented with 6.0 μg/ml chloramphenicol, 50 μl 20 mg/ml X-gal (Bio Basic, Amherst, NY, USA), and 10 μl 1.0 M IPTG (Bio Basic). Furthermore, β-galactosidase activity was measured using Miller’s (1972) method. One unit of β-galactosidase activity was defined as the amount of enzyme required to produce 1.0 μmol of o-nitrophenol per min.

## RESULTS

### CONSTRUCTION OF pKW2124-DERIVED VECTORS

**FIGURE 1:**
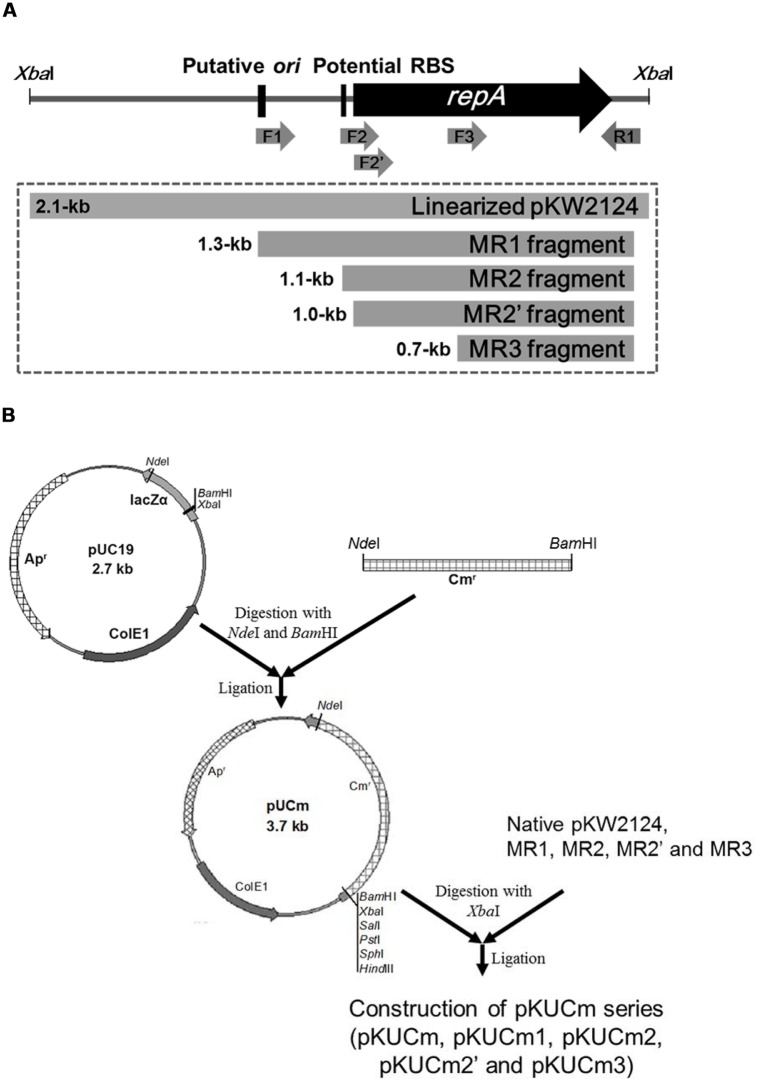
**Preparation of PCR-amplified potential *Weissella* minimal replicons and their cloning into pUCm vector. (A)** Four different PCR products (MR1, MR2, MR2’, and MR3) were obtained to determine the minimal replicon of pKW2124. **(B)** The entire pKW2124, MR1, MR2, MR2’, and MR3 products were cloned into pUCm (pUC19+CAT gene), resulting in pKUCm, pKUCm1, pKUCm2, pKUCm2’, and pKUCm3, respectively. F1, F2, F2’, F3, and R1 indicate the WC-F1-XbaI, WC-F2-XbaI, WC-F2’-XbaI, WC-F3-XbaI, and WC-R1-XbaI PCR primers, respectively.

The entire pKW2124 plasmid sequence contains a plasmid replication cluster consisting of a putative origin of replication (*ori*), a potential ribosomal binding site (RBS), and a *repA* gene encoding a plasmid replication protein (Kim et al., 2013). To elucidate their functions and to determine the minimal replicon of pKW2124, four different PCR amplicons of pKW2124 (MR1, *ori*+RBS+*repA*; MR2, RBS+*repA*; MR2’, *repA*; MR3, fragment of *repA*) were prepared with different oligonucleotide primer sets containing *Xba*I for cloning (**Figure 1A**). For efficient selection of gram-positive bacterial transformants, the 1.3-kb *cat* gene from the *Staphylococcus aureus* plasmid pC194 was introduced into the *Nde*I-*Bam*HI sites of pUC19, resulting in the 3.7-kb vector, pUCm. A linearized whole plasmid pKW2124 by *Xba*I restriction and each PCR amplicon (MR1, MR2, MR2’, and MR3) were cloned into the *Xba*I-site of pUCm, respectively, forming the recombinant plasmids, pKUCm (5.8-kb), pKUCm1 (5.1-kb), pKUCm2 (4.8-kb), pKUCm2’ (4.7-kb), and pKUCm3 (4.4-kb; **Figure 1B**).

### DETERMINATION OF THE FUNCTIONAL MINIMAL REPLICON AND TRANSFORMATION EFFICIENCY

In order to verify the minimal replicon, the pKUCm series of vectors were introduced into *W. confusa* ATCC 10881 by electroporation, and transformants were selected using selective agar plates containing chloramphenicol. The electroporation efficiencies of pKUCm containing the entire pKW2124 plasmid and pKUCm1 were similar, suggesting that the MR1 region containing a putative *ori* region, RBS, and RepA should be required for optimum plasmid replication (**Table 2**). In addition, the electroporation efficiency of pKUCm2 was extremely low, even though the RBS and RepA were present in pKUCm2, indicating that the putative *ori* region is the key component for plasmid replication (**Table 2**). To confirm the role of RBS in *Weissella* plasmid replication, the vector pKUCm2’ [pUCm+MR2’ (only Rep protein without RBS)] was constructed with PCR-amplified MR2’ region with a forward primer (WC-F2’-XbaI) and a reverse primer (WC-R1-XbaI; **Table 1**). This constructed vector, pKUCm2’ vector was transformed into *Weissella* host. However, no colony was detected even after 3 days incubation at 37°C, suggesting that RBS is an essential component for *Weissella* plasmid replication. The electroporation of pKUCm3 into *W. confusa* host showed no transformants, probably due to a lack of components required for plasmid replication (**Table 2**). Based on the electroporation results, the MR1 region is the minimum replicon of plasmid pKW2124. This minimal MR1 replicon was used for further construction of a *Weissella* expression vector with optimum transformation efficiency.

**Table 2:** Transformation efficiency of different pKUCm series vectors with the *W. confusa* ATCC 10881 host.

pKUCm series vector	Transformation efficiency (CFU μg^--1^ DNA)
pKUCm (Whole pKW2124)	1.9 × 10^5^
pKUCm1 (*ori*+RBS+*repA*)	1.5 × 10^5^
pKUCm2 (RBS+*repA*)	1.3 × 10^1^
pKUCm2’ (*repA*)	No colony
pKUCm3 (disrupted *repA*)	No colony

**FIGURE 2:**
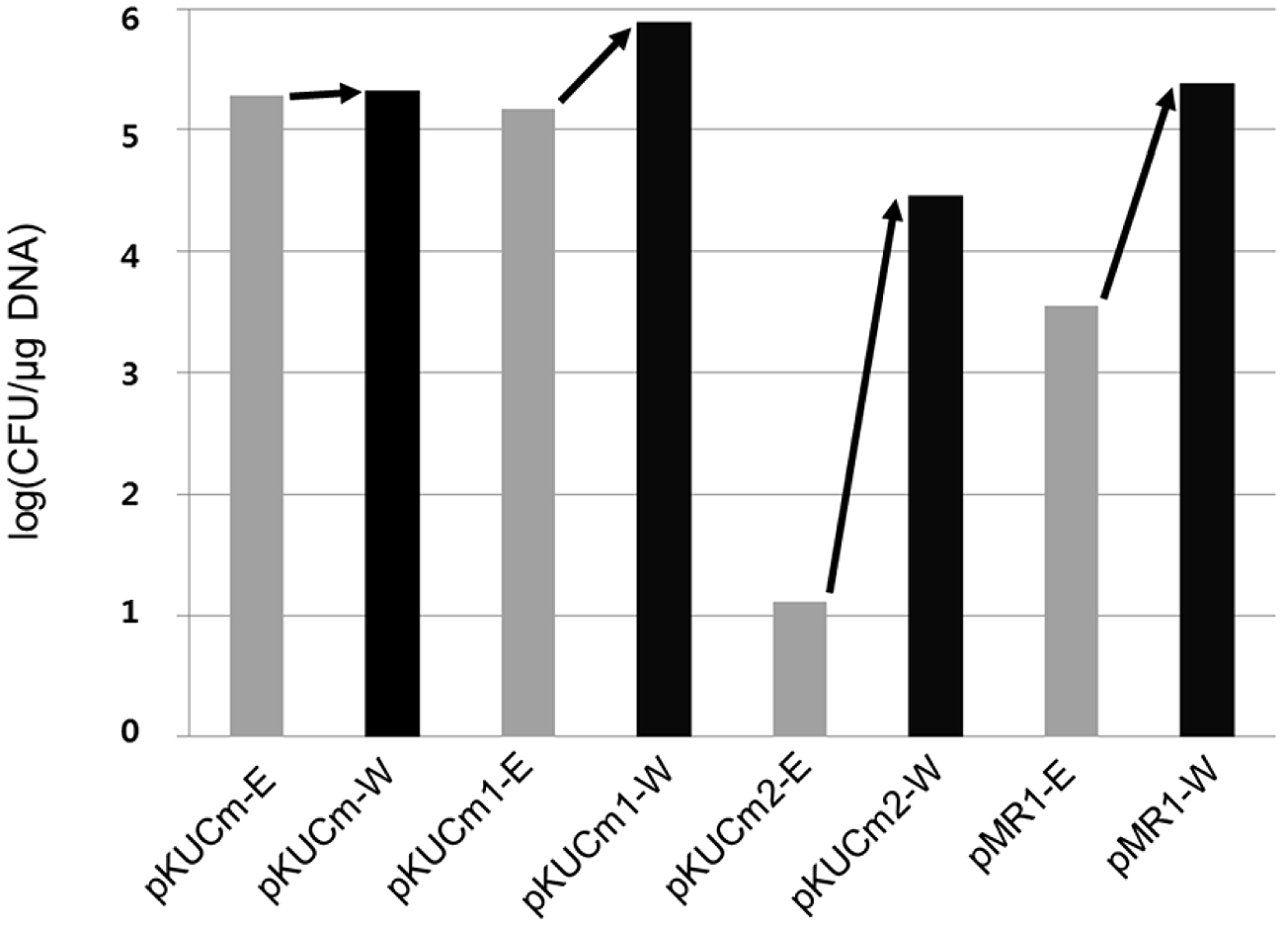
**Evaluation of pKUCm series and pMR1 vector re-transformation efficiencies from *W. confusa* ATCC 10881 into the same strain (black scale bar), compared with the vectors prepared from *Escherichia coli* DH5α into *W. confusa* ATCC 10881 (gray scale bar).** Re-transformation of the vectors from *Weissella* improved the transformation efficiency.

In this study, the transformation efficiency of the pKUCm series of vectors showed that pKUCm vectors containing the complete MR1 region (pKUCm and pKUCm1) were easily used to stably transform hosts via electroporation. However, the pKUCm vectors containing a partial MR1 region or lacking the MR1 region (pKUCm2 and pKUCm3, respectively) were highly unstable or did not replicate in the host. While these transformation efficiencies were studied with the vectors in *E. coli*, we confirmed these pKUCm series vector transformation efficiencies between *Weissella* hosts using vectors isolated from *Weissella* transformants to re-transform *Weissella* hosts. Interestingly, the re-transformation efficiencies increased (**Figure 2**). In particular, the re-transformation efficiency of pKUCm2, which showed very low transformation efficiency from *E. coli* to *Weissella*, was strikingly increased by more than 10^3^ times. While the reason for improvement of transformation efficiency during re-transformation between *Weissella* hosts is not clearly understood at this time, it may be due to the restriction–modification (R–M) system of the *Weissella* host.

To confirm MR1 as a minimal plasmid replicon, the MR1 and CAT gene were obtained from pKUCm1 vector using PCR with a forward primer (pMR1-F-PstI) and a reverse primer (pMR1-R-PstI). The PCR product was digested with *Pst*I and self-ligated, resulting in a 2.7-kb pMR1 (**Table 1**). The vector pMR1 was electroporated into *W. confusa* host, showing a transformation efficiency of 3.6 × 10^3^ CFU/μg DNA, substantiating that MR1 is a *Weissella* plasmid minimal replicon. Interestingly, subsequent electroporation of pMR1 after recovery from the selected *W. confusa* transformant showed approximately 67-fold increment of its transformation efficiency (2.4 × 10^5^ CFU/μg DNA), probably due to the host R–M system (**Figure 2**).

### SEGREGATIONAL PLASMID STABILITY

**FIGURE 3:**
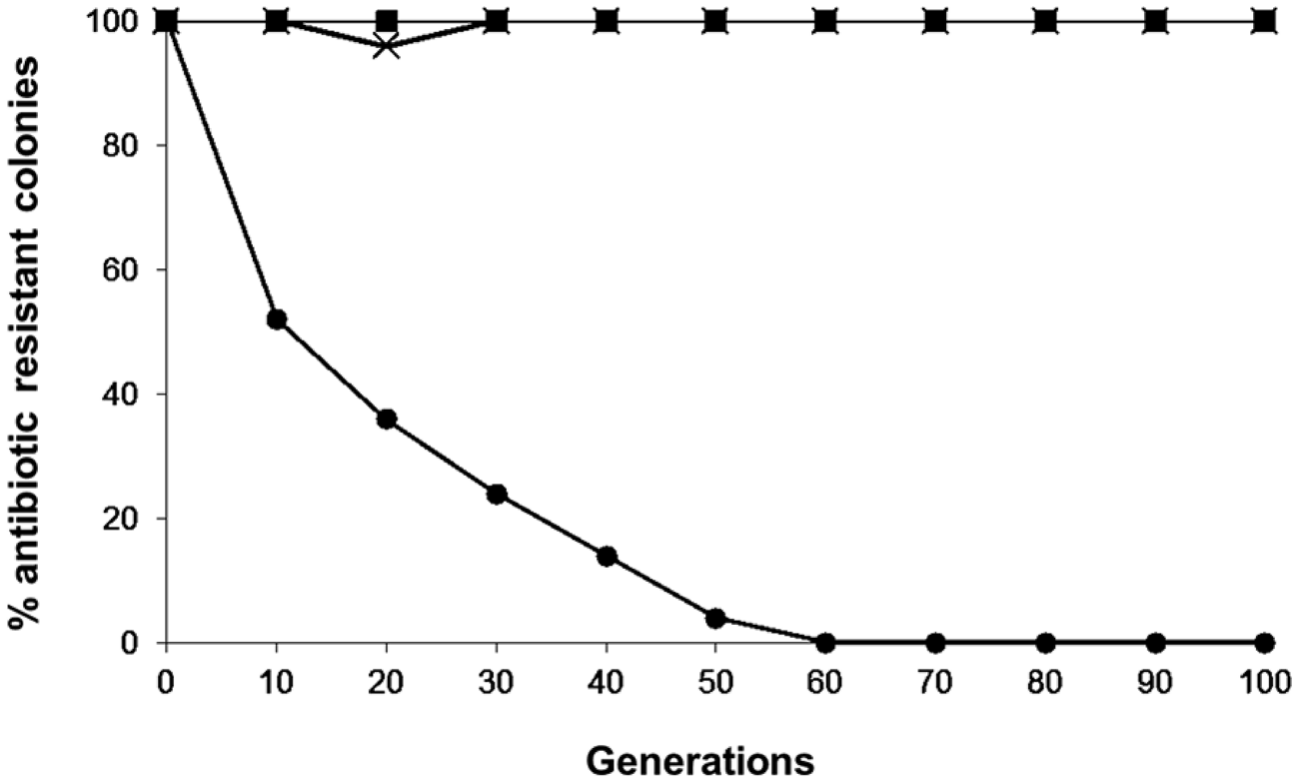
**Segregational stability of pKUCm, pKUCm1, and pKUCm2 in *W. confusa* ATCC 10881.** The presence of plasmids was verified by plasmid preparation and restriction digest analysis followed by gel electrophoresis. Symbols: pKUCm, (■); pKUCm1, (X); pKUCm2, (●).

The plasmid stability results of the pKUCm, pKUCm1, and pKUCm2 vectors in their transformants are illustrated in **Figure 3**. In the absence of antibiotic selective pressure, nearly 100% of the transformants retained the pKUCm and pKUCm1 vectors for up to 100 generations, most likely because both of these vectors contain the MR1 region for optimum plasmid replication and plasmids containing the MR1 from pKW2124 are replicated by theta replication (Kim et al., 2013). Previous segregational plasmid stability results of pKW2124 also support this finding. However, plasmid stability results of pKUCm2 revealed that this vector is unstable in the transformant, most likely due to a lack of the putative *ori* region, resulting in the complete loss of the vector after 60 generations. This segregational plasmid stability test also substantiated the fact that the MR1 region is required for optimal plasmid replication.

### HOST RANGE OF pKUCm1

To investigate the replication ability of pKUCm1 in various host strains, the vector was introduced into the closely related LAB and *Bifidobacterium* strains listed in **Table 1**. The pKUCm1 plasmid was able to replicate in different genera, including *Leuconostoc citreum, Lactococcus lactis, Lactobacillus plantarum,* and *W. paramesenteroides*, although their transformation efficiencies were very low (**Table 3**), and pKUCm1 could not replicate in the *Streptococcus thermophilus* and *Bifidobacterium bifidum* species (**Table 3**). Subsequent transformation efficiencies of pKUCm1 vector recovered from each selected LAB transformant into the same host competent cells were not quite different from the first transformation efficiencies, but slightly increased, supporting the predicted contribution of plasmid transformation efficiency and stability by the host R–M system (**Table 3**). This result suggests that pKUCm1 is a broad-host range vector and may be able to be used as a molecular tool in LAB.

**Table 3:** Transformation efficiency of pKUCm1 with selected LAB and *B. bifidum* hosts.

Bacterial strain	Transformation efficiency (CFU μg^--1^ DNA)^a^	
	First transformation	Second transformation
*Leuconostoc citreum* 75X	2.7 × 10^0^	3.7 × 10^0^
*Lactococcus lactis* MG1363	1.3 × 10^0^	1.3 × 10^0^
*Lactobacillus plantarum* 50W	1.0 × 10^0^	1.7 × 10^0^
*W. paramesenteroides* KCTC 3531	5.3 × 10^0^	7.7 × 10^0^
*Streptococcus thermophilus* ATCC 19258	No colonies	–
*B. bifidum* ATCC 15521	No colonies	–

*^a^First transformation indicates the transformation of pKUCm1 from the selected *E. coli* transformant into each LAB competent cell; Second transformation indicates the re-transformation of pKUCm1 from each selected LAB transformant into the same host competent cell*.

### ANALYSIS OF THE *W. cibaria* KSD1 β-GALACTOSIDASE GENE AND PROMOTER REGION

**FIGURE 4:**
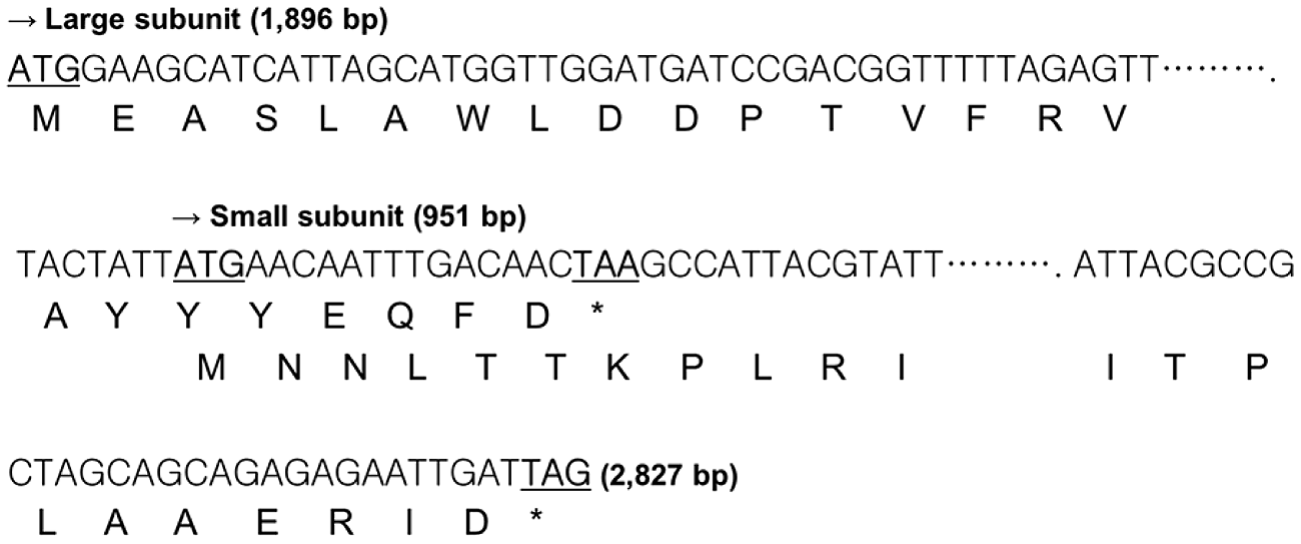
**Overlapping coding region of the *W. cibaria* KSD1 β-galactosidase gene large and small subunits.** The asterisk means stop codon.

**FIGURE 5:**
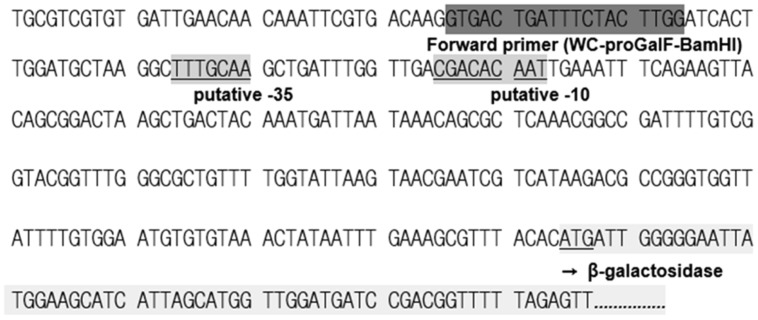
**Putative promoter region of the *W. cibaria* KSD1 β-galactosidase gene.** The promoter region was predicted using the BPROM program.

To verify that the pKUCm1 can be used as a gene expression vector, we cloned and expressed the β-galactosidase gene. The DNA sequence of the *lacZ* gene encoding β-galactosidase was obtained from the genome sequence of *W. cibaria* KSD1 (Jung et al., Unpublished data). The *W. cibaria* KSD1 β-galactosidase was encoded by two overlapping genes, a large subunit gene (1,896-bp encoding 636 amino acids) and a small subunit gene (951-bp encoding 316 amino acids). The large subunit gene is located upstream of the small subunit gene and the two genes overlap by 20-bp nucleotides (**Figure 4**). The putative -35 and -10 regions were predicted using the BPROM program (Li, 2011), which are located approximately 200-bp upstream of the start codon for the large subunit (**Figure 5**). Based on these results, two forward and reverse primers, WC-proGalF-BamHI and WC-proGalR-BamHI, were chemically synthesized for PCR amplification of the *W*. *cibaria* KSD1 β-galactosidase gene containing its promoter region (**Table 1**).

### β-GALACTOSIDASE GENE EXPRESSION USING pKUCm1 AND DETERMINATION OF ENZYME ACTIVITY

**FIGURE 6:**
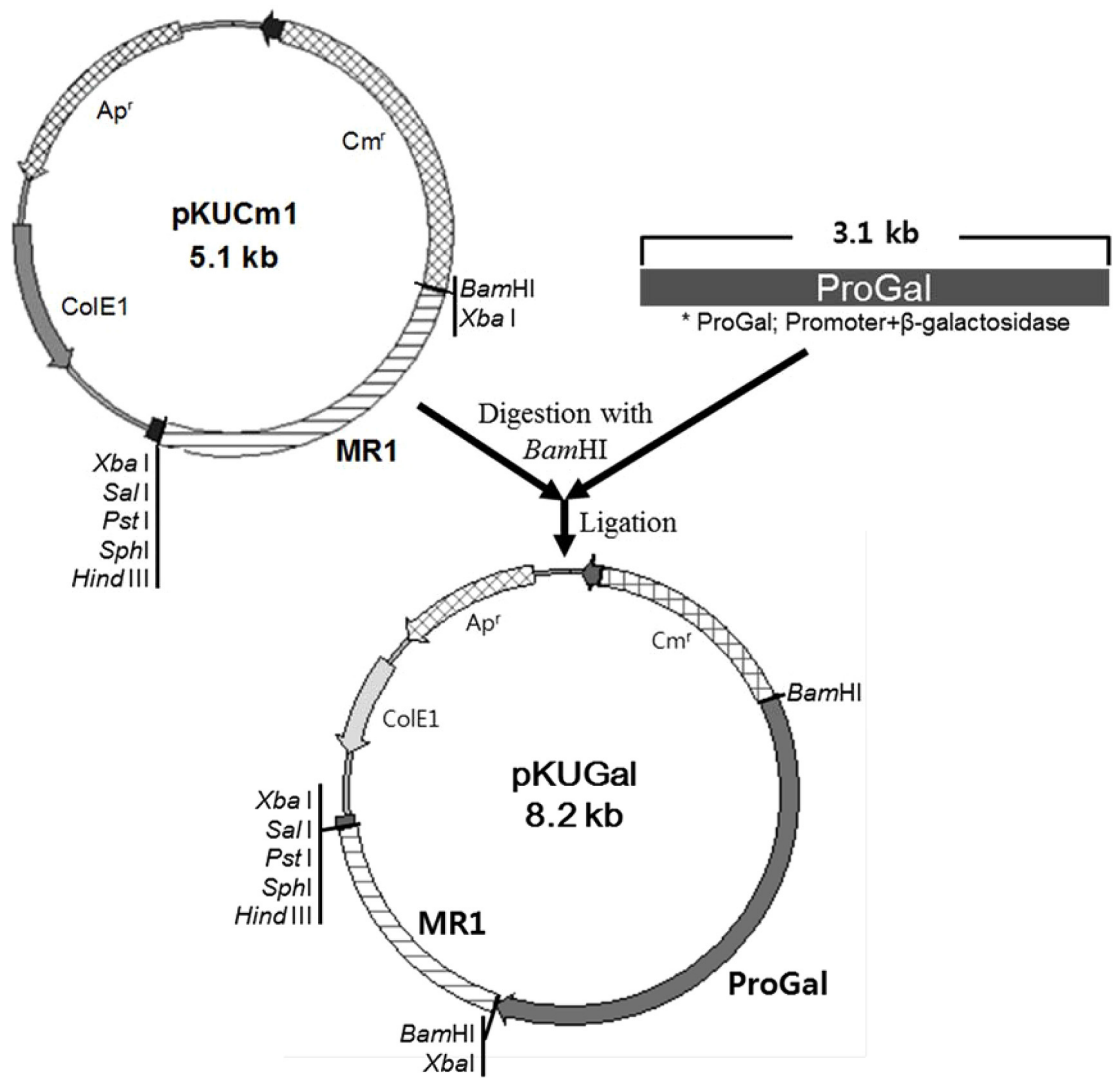
**Construction of the cloned pKUGal vector for β-galactosidase gene expression.** The β-galactosidase gene containing the putative promoter region (ProGal) was cloned into the *Bam*HI site of pKUCm1.

**FIGURE 7:**
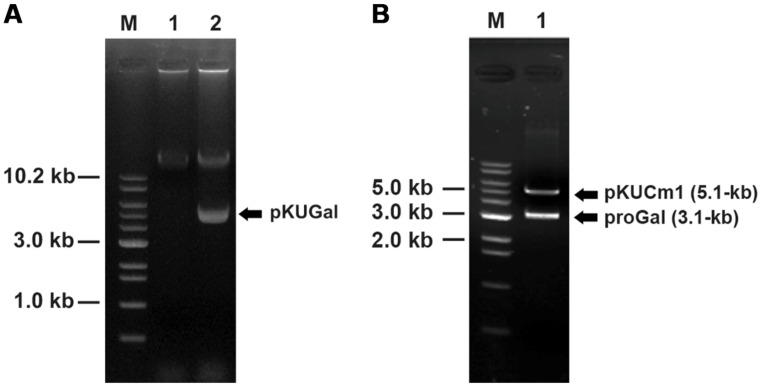
**Confirmation of vector introduction into *W. confusa* ATCC 10881 using restriction analyses followed by gel electrophoresis. (A)** Lane 1, Wild-type *W. confusa* ATCC 10881; Lane 2, *W. confusa* ATCC 10881 harboring pKUGal; M, 1.0 kb DNA ladder (Bioneer). **(B)** gel-extracted pKUGal (8.2-kb) digested to 5.1-kb pKUCm1 and 3.1-kb ProGal by *Bam*HI.

**FIGURE 8:**
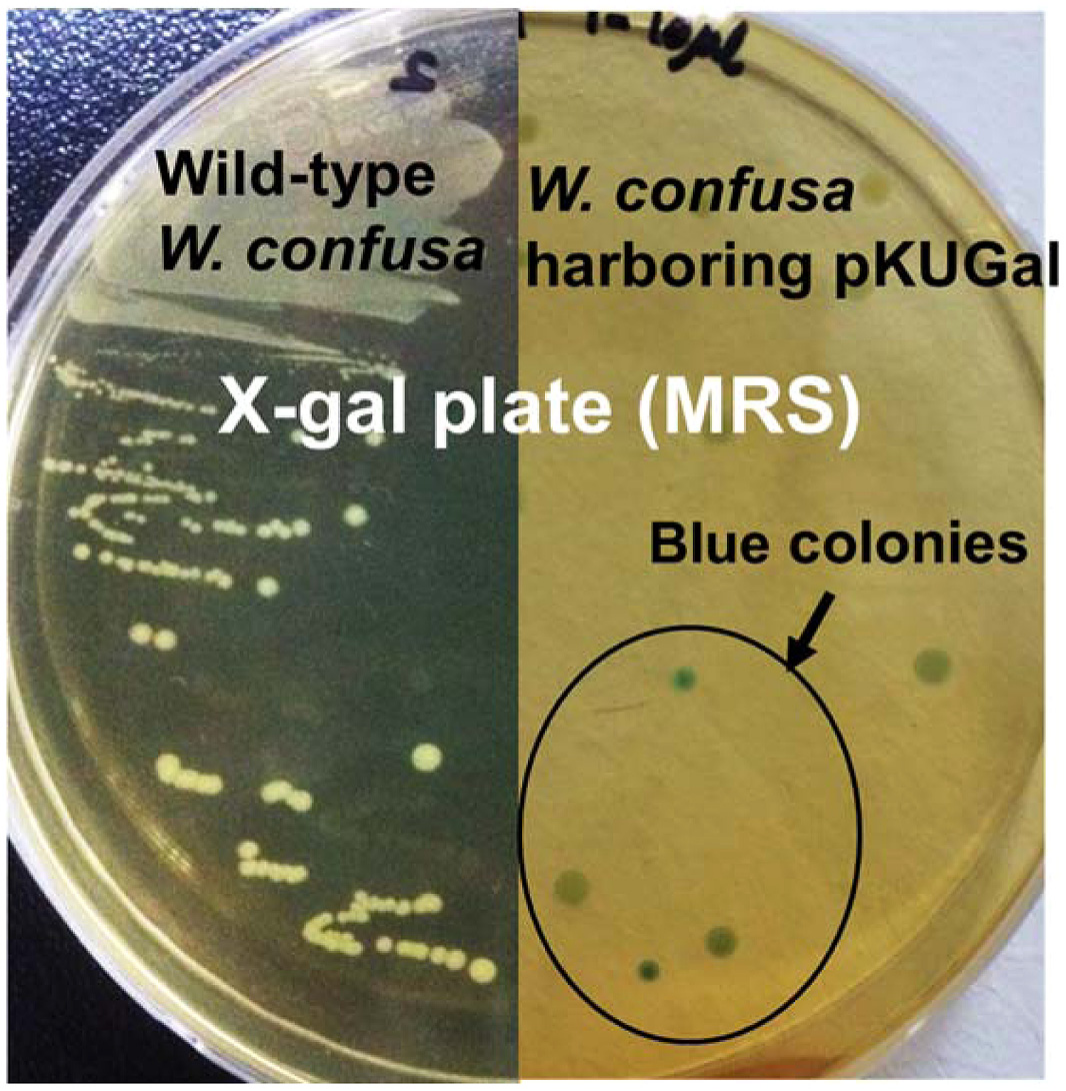
**β-galactosidase activity of *W. confusa* ATCC 10881 harboring pKUGal using blue-white screening.** Only transformants harboring pKUGal formed blue colonies, indicating that the transformants produce β-galactosidase from the introduced vector. Wild-type *W. confusa* ATCC 10881 forming white colonies were used as a negative control.

The *W. cibaria* KSD1 β-galactosidase gene containing its native promoter region was PCR-amplified to generate a 3.1-kb PCR product. The PCR product was purified and cloned into the *Bam*HI site of pKUCm1, forming the recombinant plasmid pKUGal (**Figure 6**), which was then introduced into *W. confusa* ATCC 10881 by electroporation. Selected transformants containing pKUGal were confirmed by plasmid re-isolation and restriction enzyme digestion analysis (**Figure 7**). In addition, β-galactosidase production by the selected transformant harboring pKUGal was confirmed using the blue-white screening method. After incubation of the selected transformant on MRS agar containing X-gal and IPTG, blue colonies were isolated while the wild-type *W. confusa* ATCC 10881 did not show blue colonies (**Figure 8**). To determine the enzyme activity of β-galactosidase, the selected transformant containing pKUGal and the wild-type *W. confusa* ATCC 10881 negative control were incubated for 10 h. After incubation and cell disruption, the supernatant of the negative control did not show any enzyme activity for lactose utilization, while the supernatant of the selected transformant showed an enzyme activity of 0.34 U/ml, confirming that the selected transformant produced intracellular β-galactosidase.

## DISCUSSION

The genus *Weissella* has been frequently detected and isolated from various fermented foods worldwide (except for *W. soli* that was isolated from a soil sample; Magnusson et al., 2002; Lee et al., 2010), indicating that *Weissella* species may play an important role in food fermentation. The *Weissella* genus has been recognized as a potential probiotic due to its frequent detection and isolation in foods; however, Fairfax (2014) recently suggested that *W. confusa* may be linked to infectious diseases in humans and animals, potentially causing sepsis and other serious infections, implying that use of *W. confusa* may need careful consideration for probiotic applications. Nevertheless, the probiotic study of various *Weissella* species has focused on human health-promoting properties such as antimicrobial activity against pathogens (Cai et al., 1998), inhibition of adherence of *H. pylori* (Nam et al., 2002), and reduction of volatile sulfur compound (VSC) production involved in the oral cavity (Kang et al., 2006). In particular, this genus is one of the most dominant microbes in traditionally Korean fermented foods including kimchi, makgeolli, and jeotgal, to name a few (Kim and Chun, 2005; Guan et al., 2011; Min et al., 2012). Molecular studies will need to be done to explore the health promoting effects of the genus *Weissella*.

To date, only four *Weissella* plasmids including pKLCA, pKLCB, pKLCC, and pKW2124 have been isolated and studied at the molecular level (Park et al., 2007; Kim et al., 2013). Among them, pKW2124 was replicated using the theta replication mechanism and is extremely stable in selected transformants (Kim et al., 2013). Therefore, this plasmid was used for the construction of a cloning and expression vector, pKWCSLGFP (8.6-kb), which was tested by the cloning and expression of the *gfp* gene. This vector successfully expressed and produced GFP protein as a surface display vector in the genus *Weissella* (Kim et al., 2013). However, this vector required optimization by minimizing the *Weissella* plasmid pKW2124 replicon for easy handling and better transformation efficiency. To determine the minimal replicon of the *Weissella* plasmid, four different plasmid regions (MR1, MR2, MR2’, and MR3) were obtained by PCR amplification. After cloning and transformation of pKUCm (whole plasmid pKW2124), pKUCm1 (MR1), pKUCm2 (MR2), pKUCm2’ (MR2’), and pKUCm3 (MR3), the minimal replicon of pKW2124 was determined to be a putative *ori* region, an RBS, and the *repA* gene as essential components for plasmid replication. Previous studies that minimized plasmid replicons in other genera including *Leuconostoc, Bifidobacterium,* and *Lactobacillus*, demonstrated that the minimal replicons consist of these same three essential components (Cronin et al., 2007; Yin et al., 2009; Chae et al., 2013). Furthermore, transformation efficiencies of pKUCm and pKUCm1 were not different, substantiating the fact that the MR1 region contains the minimal replicon for pKW2124 replication. Using the MR1 region, the minimized cloning vector pKUCm1 was further used for specific gene expression.

Actually, the vector pKUCm1 contains two replicons, ColE1 from pUC19 and *Weissella* plasmid replicon from pKW2124. Therefore, it may be important to clarify which plasmid replicon really works for *Weissella* host. The additional experiment to clarify this, the vector pUCm without *Weissella* plasmid replicon was chosen and electroporated into *W. confusa*. However, no colony was detected on the selective agar plates containing chloramphenicol, substantiating that the ColE1 replicon in pUCm vector does not work for plasmid replication in *Weissella* host (data not shown). In addition, the results of pUCm (pUC19+CmR), pKUCm2 [pUCm+MR2 (without plasmid *ori* of *Weissella*)], pKUCm3 [pUCm+MR3 (with only fragmented Rep protein)] in **Table 2** were disappointing for cloned vector replications in *Weissella*, substantiating that only ColE1 replicon cannot replicate the vector replication. It was previously reported that the ColE1 replicon is one of the narrow-host-range replicons like pSC101 (Kües and Stahl, 1989) and it requires only replication proteins from *E. coli* host (Tomizawa et al., 1977; Donoghue and Sharp, 1978). To confirm this, Diaz and Staudenbauer (1982) showed that ColE1 replicon cannot replicate in cell-free *Pseudomonas* extracts but it showed a partial replication ability when purified *E. coli* gyrase and DNA polymerase I are added, substantiating this narrow host range of ColE1 replicon. However, ColE1 replicon in pKUCm1 may still need to make cloning of specific gene easier in *E. coli* host than in *Weissella* host. To verify the contribution of the ColE1 replicon for plasmid replication in *Weissella*, only Cm^r^ and *Weissella* minimal replicon regions in pKUCm1 vector were amplified by PCR and self-ligated. And then the ligated plasmid, designated 2.7-kb pMR1, was electroporated into the *Weissella* host strain. Additional transformation experiment into *W. confusa* host strain with pMR1 showed lower transformation efficiency (3.6 × 10^3^ CFU/μg DNA) than that of pKUCm1 vector (1.5 × 10^5^ CFU/μg DNA), indicating that the elements of ColE1 replicon may contribute to replication of pKUCm1 vector in *Weissella*. After recovery of pMR1 from the selected *W. confusa* transformant, it was re-transformed into the *W. confusa* competent cells, showing the increment of transformation efficiency of pMR1 in *Weissella* (2.4 × 10^5^ CFU/μg DNA). However, this enhanced transformation efficiency of pMR1 is still slightly lower than that of re-transformed pKUCm1 vector (7.8 × 10^5^ CFU/μg DNA), suggesting that contribution of the ColE1 replicon to the plasmid replication in *Weissella* may be still valid even in the re-transformation into *Weissella*, even though the host R–M system may be involved in this increment of the transformation efficiency.

For expression of *lacZ* gene, the putative promoter region (TTGTGG as putative -35 box and TATAAT as putative -10 box) was initially found upstream *lacZ* gene of *W. cibaria* KSD1. To amplify this *lacZ* gene containing the putative promoter region using PCR, a primer set including a forward primer (5′-GGATCCCCGGGTGGTTATTTTGTGGA-3′) and a reverse primer (WC-proGalR-BamHI in **Table 1**) was designed and chemically synthesized. This forward primer contains putative -35 box (TTGTGG, indicated in the forward primer with underbar). The insert DNA containing *lacZ* gene and its putative promoter region was obtained by PCR with this primer set and cloned into pKUCm1 expression vector. However, there was no blue colony on selective X-gal plate, indicating that this gene was not expressed in the vector system, probably due to wrong promoter region. To detect the other candidate of consensus sequences as -35 and -10 boxes, the BPROM program was used for prediction of bacterial promoters (Li, 2011) and the other possible promoter region was detected (**Figure 5**). And then, the *Weissella lacZ* gene including this newly detected promoter region was amplified by PCR and cloned into the expression vector pKUCm1, designated pKUGal. Finally, we detected blue colonies on selective X-gal plate, substantiating that newly detected promoter region really works for binding of ribosome to translate mRNA of β-galactosidase (**Figure 8**).

In the previous study, the surface display vector pKWCSLGFP was constructed using pKW2124 and was used for *gfp* gene expression (Kim et al., 2013). The advantage of the pKWCSLGFP vector is due to specific gene expression and secretion of the produced protein. In this study, the novel expression vector pKUCm1 was constructed using the minimal pKW2124 replicon that can be used for the expression of the β-galactosidase gene for intracellular production. Based on these two vector systems, a new *Weissella* expression/secretion vector system combining the advantages of these two vectors could be developed as a future goal. Furthermore, strong promoters and inducible promoters need to be studied to improve levels of gene expression in this *Weissella* vector system. A version of pKUGal without its own promoter could be developed and used for promoter screening in *Weissella*.

This newly developed expression vector system showed high stability in the host strains as well as specific gene expression and protein production. Therefore, this vector system could be used for specific gene delivery/expression and production of protein in *Weissella*. In addition, this vector system may be useful for the elucidation of specific *Weissella* gene functions as a molecular genetic tool via cloning/expression or knockout of specific *Weissella* genes. This is the first report of a plasmid minimal replicon and its application for development of an improved vector system in *Weissella*.

## Conflict of Interest Statement

The authors declare that the research was conducted in the absence of any commercial or financial relationships that could be construed as a potential conflict of interest.
